# Characteristics, Antecedents, and Consequences of Non-Nursing Tasks: A Scoping Review Protocol

**DOI:** 10.3390/nursrep15050153

**Published:** 2025-04-30

**Authors:** Gaia Magro, Erika Bassi, Stefania Chiappinotto, Gaia Dussi, Elisa Ambrosi, Federica Canzan, Marco Clari, Alessio Conti, Alberto Dal Molin, Alvisa Palese

**Affiliations:** 1 Department of Medicine, University of Udine, 33170 Udine, Italy; magro.gaia@spes.uniud.it (G.M.); stefania.chiappinotto@uniud.it (S.C.); dussi.gaia@spes.uniud.it (G.D.); alvisa.palese@uniud.it (A.P.); 2 Department of Translational Medicine, University of Piemonte Orientale, 28100 Novara, Italy; alberto.dalmolin@med.uniupo.it; 3 Department of Diagnostics and Public Health, University of Verona, 37134 Verona, Italy; elisa.ambrosi_01@univr.it (E.A.); federica.canzan@univr.it (F.C.); 4 Department of Public Health and Pediatrics, University of Torino, 10126 Torino, Italy; marco.clari@unito.it; 5 Department of Clinical and Biological Sciences, University of Torino, 10043 Torino, Italy; alessio.conti@unito.it

**Keywords:** non-nursing tasks, nonvalue-added care, nurses, nursing staff, scoping review

## Abstract

**Background**: Nurses report that they often must perform activities outside their area of expertise, referred to as “non-nursing tasks”. The time spent on simple tasks undermines nursing capacity, which is already challenged by the dramatic shortage of nurses. Performing non-nursing tasks affects nurses’ satisfaction and their intention to stay. However, despite their relevance, there is no summary of studies on non-nursing tasks. The aim of this study is to summarize available evidence on characteristics of non-nursing tasks, their antecedents, and consequences, to identify gaps in the existing literature and to make recommendations for management, education, practice, and nursing research. **Methods**: A scoping review will be conducted using Arksey and O’Malley’s methodological framework. The Preferred Reporting Items for Systematic reviews and Meta-Analyses extension for Scoping Reviews, and the Patterns, Advances, Gaps, Evidence for practice and research framework will be used to report and summarize the findings. **Results**: Findings will provide a map regarding the main patterns of research in this area and the evidence produced to date. Two main lines of findings are expected, namely the conceptual and the empirical. The former may contribute to the understanding of the terminology and concept used so far to clearly express non-nursing tasks; the empirical evidence may shed light on frequencies, instruments, reasons, and effects. **Discussion**: An analysis of non-nursing tasks will provide healthcare organizations with a conceptual framework of all variations in the phenomenon and will help managers to identify the activities that will fall within this conceptual construct. The establishment of a research strand in this field, based on a science-based review involving international stakeholders, can strengthen global action to prevent the occurrence of non-nursing tasks.

## 1. Introduction

Nurses are expected to carry out their role within the scope of their discipline. However, in various situations, to compensate for organizational deficiencies, they may not make full use of their competencies or perform activities for which they have not been adequately trained [1]. This phenomenon has now sparked renewed interest: on the one hand, the appropriate use of nursing competencies can increase the quality of care [2], and on the other hand, it can also mitigate the nursing shortage, as this is due to the proportion of time nurses spend on activities outside their scope of expertise, the so-called “non-nursing tasks” [3] (NNTs) phenomenon.

NNTs have been defined as interventions that are not directly related to patient care [4] and that do not require nurses to apply the knowledge acquired in their education or to make clinical judgment. This phenomenon affects nurses worldwide [5] and accounts for between 35% [6] and 62% of the duration of a nursing shift [7,8]. According to the available literature, NNTs reflect three categories of activities: activities that require lower levels of education (e.g., activities expected of nursing assistants), activities that require the same level of education as nurses but in other fields (e.g., activities expected of other healthcare professionals), and activities that require higher education (e.g., activities expected of physicians) [9]. Closely following the concept of NNTs, non-value-added activities [10] include all tasks potentially falling to nursing assistants or administrative staff, e.g., locating equipment, carrying stable patients, and completing paperwork [8,11,12]. All tasks expected of low-skilled health workers, as well as so-called non-value-added activities, have been considered a concern at the policy level, as they may waste nurses’ competencies, further limit their time with patients, exacerbate nurse shortages, and lead to role ambiguity that prevents nursing programs from being attractive [13].

Overall, several reasons, factors, or antecedents (hereinafter, antecedents) for NNTs have recently been identified, both at the organizational level, such as the revision of skill mix and the increase in organizational work, and at the nurse level, such as the desire to meet patients’ needs and ensure their comfort and well-being under all critical organizational conditions [8,14]. In addition, nursing education and professional socialization, which shape professional identity, have been recognized as antecedents of NNTs [3]. The consequences of NNTs have also been reported, with effects identified at different levels, namely (a) at the organizational level, such as poor quality of care [15] and increased unfinished care [16]; (b) at the nurse level, such as a diminished sense of autonomy [15], perceived wasted time, frustration at no longer being in the role of nurse [17], and tensions with other healthcare workers [3]; and (c) at the patient level, such as an increased risk of receiving poor care [9,18]. In line with the World Health Organization’s call to develop strategies to address the dramatic shortage in nurses, estimated to be approximately 4,5 million by 2030 [19], the adequacy of the role of nurses should be high on the agenda of international policy and research. However, there is no summary of primary studies on the prevalence of NNTs and their key antecedents that could serve as a basis for effective action. Furthermore, there is no summary of the consequences of NNTs to identify their impact on patients, nurses, and organizations.

### The Aim of the Study

The overall aim of this study is to explore the patterns in existing research on NNTs or non-value-added activities (hereafter: non-nursing tasks). Specific aims are to summarize the available evidence on the characteristics of NNTs, their antecedents, and consequences; to identify gaps in existing literature; and to make recommendations for (a) management, (b) education, (c) practice, and (d) nursing research.

## 2. Materials and Methods

A six-step scoping review study will be conducted according to the available guidelines by Arksey and O’Malley [20], which have been updated in their methodology [21,22,23]. To ensure rigor, additional methodological approaches will also be applied. In particular, the Population, Concept, and Context (PCC) framework will be used to define the components of the review question and guide the literature search [20,24,25]. In addition, the Preferred Reporting Items for Systematic reviews and Meta-Analyses extension for Scoping Reviews (PRISMA-scr) will be followed to promote the quality of the review process [23,26]. Moreover, the Patterns, Advances, Gaps, Evidence for practice and Research (PAGER) recommendation framework will be used for data presentation and synthesis to identify (a) the patterns that have emerged in the available research, (2) the advances that have been made to date, (3) the gaps that remain in the available studies, and (4) the evidence for practice and research recommendations [21]. Finally, reflexivity will be promoted through the effective involvement of the research team (see authors) in multiple sessions. The protocol for this review has been registered by the Ricerca Infermieristica Clinica e per l’Innovazione organizzativa e formativa (RICI) group research team (Table S1) with the Open Science Framework https://doi.org/10.17605/OSF.IO/JUSCR (accessed on 19 November 2024). No ethical approval is required for this study as the scoping review methodology involves the review and collection of data from publicly available material.

### 2.1. Step 1: Identifying the Research Question

The study addresses three central research questions: (1) “What terminologies are used to describe NNTs?” (2) “How are NNTs conceptualized and defined?” (3) “What main features, antecedents, and consequences of NNTs are documented in the available literature to date?”. According to the PCC framework [20,24,25], the focus is on P = nurses; C = concept of NNTs; and C = any work context in which nurses are involved, such as hospital, community, and intermediate care settings.

### 2.2. Step 2: Identifying Relevant Studies

An initial search of the MEDLINE database (via PubMed) was conducted by the first author in November 2024 to identify subject headings and keywords to refine the search strategy. As a result, the following keywords and MeSH terms were selected according to the PCC framework: “nurs*”, “nursing staff”, “non-nurs* task”, “non nurs* task”, “non-nurs*”, “non nurs*”, “nurs* activit*”, “non-nurs* activit*”, “non-value task” e “non-value activit*”. To consider all settings, keywords that refer to the context (e.g., hospital) will not be included in the final search strategy. To find all studies on “NNTs” and similar concepts, the researchers will also tabulate all the different definitions they find in the search.

We will search for the following databases: PUBMED/Medline, Cumulative Index to Nursing and Allied Health Literature (CINAHL), Cochrane Database of Systematic Review, and Scopus. In addition, the reference lists of the included articles will be checked for other relevant sources. Boolean operators (AND, OR, NOT) and search functions will be used to expand or restrict the search [27]. The complete search strategy for all databases can be found in Table 1. Before the end of the review process, the search strategy will be updated to include all studies. The detailed search strategy is reported in Appendix A, Table A1.

### 2.3. Step 3: Selecting Studies to Be Included in the Review

The inclusion and exclusion criteria are based on the PCC framework to ensure a comprehensive and rigorous approach. Studies will be included if they are (1) primary quantitative or qualitative studies, mixed methods studies, or secondary studies in the form of reviews or evidence-based guidelines, (2) published in peer-reviewed journals to ensure methodological rigor and consistency, (3) focused on NNTs, and (4) written in Italian and English. Books or book chapters, commentaries, editorials, and letters will be excluded. The Rayyan program will be used to organize the retrieved sources and make the screening process more efficient [28]. In the first phase, the duplicates will be manually removed by one researcher (see first author). Subsequently, two researchers will review the eligible studies individually using the inclusion and exclusion criteria at the title and abstract level; thus, at the full-text level, two researchers will assess the studies for inclusion. A “sifting” approach will be adopted to narrow down the selection while resolving the conflicts according to Rayyan [28]. Any disagreements will be resolved through discussions with experts (see authors) who are also consulted to reach consensus. The full results of the search and the study selection process will be detailed in the final overview. A flow diagram, adhering to the PRISMA 2020 guidelines, will be used to show the number of records identified, screened, assessed for eligibility, and included in the review, and the reasons for exclusions at each stage [29]. This visual representation will improve the clarity and transparency of the selection process.

### 2.4. Step 4: Charting the Data

Data will be extracted from the studies included by two independent reviewers (see authors) using a data grid developed specifically for this scoping review. This grid, implemented as a Microsoft Excel spreadsheet, has been designed to systematically capture the detailed information related to the research questions (Table 2). The extracted preliminary grid will consider: (1) the names and affiliations of the authors, the year of publication and the country in which the study was conducted (according to the authors’ affiliation if not explicitly stated); (2) the aim of the study; (3) the study design; (4) the main study characteristics (e.g., the setting in which the study was conducted, the participants involved, the data collection instruments used and the process); (5) the concept of NNTs used (e.g., the definition given); (6) the main findings (frequency of NNTs, main characteristics); and (7) the antecedents and consequences of NNTs. In addition, further gaps and recommendations identified in each study will be extracted. The data collection grid will be tested in two studies for further refinement and the necessary changes will be made after discussion within the research team.

### 2.5. Step 5: Collating, Summarizing, and Reporting the Results

In the fifth phase, Arksey and O’Malley emphasize that the analytical framework used in scoping reviews should support the overview and presentation of a potentially large amount of material [20]. Other authors point out that it is important to understand the findings and understand the implications in a broader context, which may also have implications for policy, context, and practice [22,25]. In line with the aims of our scoping review, the PAGER framework [21] will be used to report and summarize the findings. This analytical approach will help the research team in the following steps by providing structure, showing relationships, highlighting progress and gaps, and supporting reporting: First, the patterns of the studies will be summarized by describing their main characteristics [21]; then, the advancements and evidence produced to date in terms of (a) conceptual definitions, (b) main features of the NNTs, and (c) antecedents and consequences will also be summarized. Depending on the aim of the included studies, we will summarize validated conceptual/theoretical or empirical evidence. Without restricting the publication date, it is possible that a thorough examination of the development of “NNTs” requires an organization capturing historical context, key studies, and persistent themes [30]. This approach also enables the identification of trends and shows how the concept has adapted to changes in healthcare over time.

### 2.6. Step 6: Consulting Stakeholders

At the end of the process, stakeholders at the national and international levels will be involved in the validation, interpretation, and consideration in their potential meaning for practice. A purposive sample of clinical nurses, nurse managers, nurse executives, and educators will be involved [31], as well as a purposive sample of nursing boards and patient representatives. At least one stakeholder from each group will be involved in gaining a deeper understanding of the findings and their applicability or relevance in the different settings (from education to clinical practice).

## 3. Results

The data obtained from the studies on Patterns, Advancements, and Evidence of the PAGER framework [21] will be summarized through a thematic analysis [31]. Similar findings will be grouped together to form initial codes. Based on the codes and by comparing them, broader codes will be interpretatively analyzed and merged into initial themes. The first themes will provide a condensation of information regarding the research question. Next, the data associated with each theme will be reviewed, re-examined, and analyzed to establish whether the study truly supports the themes, ensuring coherence and distinction between each theme and the others. Then, the Gaps and the Recommendations of the PAGER framework [21] will be summarized using the same process [30]. The results will be presented in tables, visual figures, and in a narrative synthesis. A PRISMA flow diagram will be presented for the study selection process [26].

## 4. Discussion

Findings will provide a map regarding the main patterns of research in this area and the evidence produced to date. Two main lines of findings are expected, namely the conceptual and the empirical. The former may contribute to the understanding of the terminology and concept used so far to clearly express NNTs; the empirical evidence may shed light on the frequencies, the instruments suitable for measuring NNTs, the reasons, and the effects. In addition, all this conceptual and empirical evidence can inform educational, organizational, and clinical practice decisions. By summarizing the evidence produced to date, this review will provide a reference point for future primary or secondary studies in this area, as well as the basis for policy development at educational and organizational levels. Summarizing the predominant types of NNTs documented in the literature will also promote discussion of the variability within the scope of nursing practice across the world, as some tasks that are considered non-nursing in certain countries and settings may be considered appropriate in others, depending on advances in the autonomy and responsibility of the nursing role. Mapping what are considered NNTs could support the interpretation of studies and policy documents on the relationship between nurses and patients at the bedside. Furthermore, by identifying gaps, this study can guide future research in accurately measuring the phenomenon and time spent on NNTs. Furthermore, by engaging stakeholders at an international level, an inclusive approach can raise awareness of the phenomenon and its key features in the broader context of the role of nursing.

### Limitations

This scoping review protocol recognizes certain limitations. First, despite the comprehensive research, it is possible that not all relevant studies will be selected due to the lack of standardized terminology and the existence of different concepts related to NNTs, such as role ambiguity or organizational work. These terms are sometimes used interchangeably, even if they have different meanings. To minimize the risk, an extensive search will be conducted in several databases. Secondly, the included studies will not be assessed for methodological quality. This lack of quality assessment could affect the conclusions of the review, as the different methodological rigors of the studies could influence the Patterns, Evidence, Gaps, and Recommendations that will be identified. In addition, the heterogeneity of study designs and methods will challenge the process of data synthesis. This heterogeneity could also make it difficult to identify consistent themes and prevent the development of a coherent narrative synthesis. However, the involvement of researchers (see authors) as well as the involvement of stakeholders will mitigate this risk.

## 5. Possible Implications of the Findings

Given the increasing shortage of nurses, providing evidence on NNTs and the potential strategies to reduce their occurrence according to documented antecedents may promote the appropriateness of nursing care; moreover, identifying the consequences may put further pressure on the need for action given the expected impact of NNTs on patients [32], nurses, and healthcare organizations. In addition, the establishment of a strong research strand in this field, based on a science-based review involving national and international stakeholders, can strengthen global action to prevent the occurrence of NNTs.

## Figures and Tables

**Table 1:** Search strategy.

Database	Query	Records 11 November 2024
PUBMED/Medline	“Nurses” [Mesh] OR “Nursing Staff”[Mesh] OR nurs* AND “non-nurs* task” OR “non nurs* task” OR “non-nurs*” OR “non nurs*” OR “nurs* activit*” OR “non-nurs* activit*” OR “non-value task” OR “non-value activit*”	2700 results
CINAHL	“nurs*” OR “nursing staff” AND “non-nurs* task” OR “non nurs* task” OR “non-nurs*” OR “non nurs*” OR “nurs* activit*” OR “non-nurs* activit*” OR “non-value task” OR “non-value activit*”	2299 results
SCOPUS	“nurs*” OR “nursing staff” AND “non-nurs* task” OR “non nurs* task” OR “non-nurs*” OR “non nurs*” OR “nurs* activit*” OR “non-nurs* activit*” OR “non-value task” OR “non-value activit*”	1462 results
COCHRANE	(nurs* OR nursing NEXT staffing OR [Nurses] OR [Nursing]) AND (non-nurs* NEXT task* OR non-NEXT nurs* NEXT task* OR non-nurs* OR non NEXT nurs* OR nurs* NEXT activit* OR non-nurs* NEXT activit* OR non-value NEXT task* OR non-value NEXT activit*)	192 results

Legend: CINAHL = Cumulative Index to Nursing and Allied Health Literature. *: to substitute one or more characters in the associated term.

**Table 2:** Data-charting grid.

ID	Authors Year Country	Study Aim(s)	Study Design	Setting, Participants, Data Collection Tools and Process	Non-Nursing Tasks				Gaps/Recommendations to Address the Future
					Conceptual Definition	Main Features (e.g., Frequency, Type)	Antecedents	Consequences	
1	…	…	…	…	…	…	…	…	…

## Data Availability

No new data were created or analyzed in this study. Data sharing is not applicable to this article.

## Supplementary Materials

The following supporting information can be downloaded at https://www.mdpi.com/article/10.3390/nursrep15050153/s1, Table S1: Members of RICI Group.

## Public Involvement Statement

No public involvement in any aspect of this research.

## Guidelines and Standards Statement

This manuscript follows the Arksey and O’Malley guidelines for scoping review [20,22,25] and the Preferred Reporting Items for Systematic Reviews and Meta-Analyses for Scoping Reviews (PRISMA-ScR) guidelines [26,29].

## Abbreviations

The following abbreviations are used in this manuscript:

NNT	Non-nursing task
PCC	Population, concept, and context
PRISMA-scr	Preferred Reporting Items for Systematic reviews and Meta-Analyses extension for Scoping Reviews
PAGER	Patterns, Advances, Gaps, Evidence for practice, and Research
CINAHL	Cumulative Index to Nursing and Allied Health Literature

## Appendix A

**Table A1:** Detailed search strategy for PUBMED/Medline, CINAHL, SCOPUS, and COCHRANE.

	Search Strategy	Results
PUBMED/Medline		
Population	“Nurses”[Mesh] OR “Nursing Staff”[Mesh] OR nurs*	1,210,108 results
Concept	“non-nurs* task” OR “non nurs* task” OR “non-nurs*” OR “non nurs*” OR “nurs* activit*” OR “non-nurs* activit*” OR “non-value task” OR “non-value activit*”	2760 results
	#1 AND #2	2700 results
CINAHL		
Population	“nurs*” OR “nursing staff”	1,005,243 results
Concept	“non-nurs* task” OR “non nurs* task” OR “non-nurs*” OR “non nurs*” OR “nurs* activit*” OR “non-nurs* activit*” OR “non-value task” OR “non-value activit*”	2329 results
	“nurs*” OR “nursing staff” AND “non-nurs* task” OR “non nurs* task” OR “non-nurs*” OR “non nurs*” OR “nurs* activit*” OR “non-nurs* activit*” OR “non-value task” OR “non-value activit*”	2299 results
SCOPUS		
Population	“nurs*” OR “nursing staff”	3,275,228 results
Concept	“non-nurs* task” OR “non nurs* task” OR “non-nurs*” OR “non nurs*” OR “nurs* activit*” OR “non-nurs* activit*” OR “non-value task” OR “non-value activit*”	3055 results
	“nurs*” OR “nursing staff” AND “non-nurs* task” OR “non nurs* task” OR “non-nurs*” OR “non nurs*” OR “nurs* activit*” OR “non-nurs* activit*” OR “non-value task” OR “non-value activit*”	1462 results
COCHRANE		
Population	nurs* OR nursing NEXT staffing OR [Nurses] OR [Nursing]	81,978 results
Concept	non-nurs* NEXT task* OR non NEXT nurs* NEXT task* OR non-nurs* OR non NEXT nurs* OR nurs* NEXT activit* OR non-nurs* NEXT activit* OR non-value NEXT task* OR non-value NEXT activit*	192 results
	(nurs* OR nursing NEXT staffing OR [Nurses] OR [Nursing]) AND (non-nurs* NEXT task* OR non NEXT nurs* NEXT task* OR non-nurs* OR non NEXT nurs* OR nurs* NEXT activit* OR non-nurs* NEXT activit* OR non-value NEXT task* OR non-value NEXT activit*)	192 results

Legend: CINAHL: Cumulative Index to Nursing and Allied Health Literature. *: to substitute one or more characters in the associated term.

## References

[1] Al Amri S.A., Dupo J., Al Kindi M.M., Al Tobi R.S., BaniOraba S.M., Torrano G., Mohammed Al Amri F., Al Aamri F.I. (2019). The Extent of Non—Nursing Tasks and Their Impact on Quality Patient Care As Perceived By Nurses in Al Dakhliyah Governorate, Oman. Saudi J. Nurs. Health Care.

[2] Aiken L.H., Sloane D., Griffiths P., Rafferty A.M., Bruyneel L., McHugh M., Maier C.B., Moreno-Casbas T., Ball J.E., Ausserhofer D. (2017). Nursing Skill Mix in European Hospitals: Cross-Sectional Study of the Association with Mortality, Patient Ratings, and Quality of Care. BMJ Qual. Saf..

[3] Palese A., Gnech D., Pittino D., Capretta F., Cossalter O., Tonet S., Pais dei Mori L., Grosso S. (2019). Non-Nursing Tasks as Experienced by Nursing Students: Findings from a Phenomenological Interpretative Study. Nurse Educ. Today.

[4] Hammad M., Guirguis W., Mosallam R. (2021). Missed Nursing Care, Non-Nursing Tasks, Staffing Adequacy, and Job Satisfaction among Nurses in a Teaching Hospital in Egypt. J. Egypt. Public Health Assoc..

[5] Ayasreh I.R., Hayajneh F., Al Awamleh R. (2022). The Impact of Performance of Non-Nursing Tasks on the Attitudes of Nursing Students toward Nursing Profession. Nurse Media J. Nurs..

[6] Fitzgerald M., Pearson A., Walsh K., Long L., Heinrich N. (2003). Patterns of Nursing: A Review of Nursing in a Large Metropolitan Hospital. J. Clin. Nurs..

[7] Bruyneel L., Li B., Aiken L., Lesaffre E., Van den Heede K., Sermeus W. (2013). A Multi-Country Perspective on Nurses’ Tasks below Their Skill Level: Reports from Domestically Trained Nurses and Foreign Trained Nurses from Developing Countries. Int. J. Nurs. Stud..

[8] Grosso S., Longhini J., Tonet S., Bernard I., Corso J., de Marchi D., Dorigo L., Funes G., Lussu M., Oppio N. (2021). Prevalence and Reasons for Non-Nursing Tasks as Perceived by Nurses: Findings from a Large Cross-Sectional Study. J. Nurs. Manag..

[9] Grosso S., Tonet S., Bernard I., Corso J., De Marchi D., Dorigo L., Funes G., Lussu M., Oppio N., Pais dei Mori L. (2019). Non-Nursing Tasks as Experienced by Nurses: A Descriptive Qualitative Study. Int. Nurs. Rev..

[10] Antinaho T., Kivinen T., Turunen H., Partanen P. (2015). Nurses’ Working Time Use—How Value Adding It Is?. J. Nurs. Manag..

[11] Michel O., Garcia Manjon A.J., Pasquier J., Ortoleva Bucher C. (2021). How Do Nurses Spend Their Time? A Time and Motion Analysis of Nursing Activities in an Internal Medicine Unit. J. Adv. Nurs..

[12] Yen P.-Y., Kelley M., Lopetegui M., Saha A., Loversidge J., Chipps E.M., Gallagher-Ford L., Buck J. (2018). Nurses’ Time Allocation and Multitasking of Nursing Activities: A Time Motion Study. AMIA Annu. Symp. Proc..

[13] Marć M., Bartosiewicz A., Burzyńska J., Chmiel Z., Januszewicz P. (2019). A Nursing Shortage—A Prospect of Global and Local Policies. Int. Nurs. Rev..

[14] Aldarawsheh A.A., Saifan A.R., Sawalha M.A., Assaf E.A., Alrimawi I., Elshatarat R.A., Saleh Z.T., Almagharbeh W.T., Mohamed N.A., Eltayeb M.M. (2024). Exploring the Causes and Consequences of Non-Nursing Tasks among Nurses in Jordan: An in-Depth Qualitative Investigation. Appl. Nurs. Res..

[15] Bekker M., Coetzee S.K., Klopper H.C., Ellis S.M. (2015). Non-Nursing Tasks, Nursing Tasks Left Undone and Job Satisfaction among Professional Nurses in South African Hospitals. J. Nurs. Manag..

[16] Chiappinotto S., Palese A. (2022). Unfinished Nursing Care Reasons as Perceived by Nurses at Different Levels of Nursing Services: Findings of a Qualitative Study. J. Nurs. Manag..

[17] Rukavina T.V., Viskić J., Poplašen L.M., Relić D., Marelić M., Jokic D., Sedak K. (2021). Dangers and Benefits of Social Media on E-Professionalism of Health Care Professionals: Scoping Review. J. Med. Internet Res..

[18] Al-Kandari F., Thomas D. (2009). Factors Contributing to Nursing Task Incompletion as Perceived by Nurses Working in Kuwait General Hospitals. J. Clin. Nurs..

[19] Boniol M., Kunjumen T., Nair T.S., Siyam A., Campbell J., Diallo K. (2022). The Global Health Workforce Stock and Distribution in 2020 and 2030: A Threat to Equity and ‘Universal’ Health Coverage?. BMJ Glob. Health.

[20] Arksey H., O’Malley L. (2005). Scoping Studies: Towards a Methodological Framework. Int. J. Soc. Res. Methodol. Theory Pract..

[21] Bradbury-Jones C., Aveyard H., Herber O.R., Isham L., Taylor J., O’Malley L. (2022). Scoping Reviews: The PAGER Framework for Improving the Quality of Reporting. Int. J. Soc. Res. Methodol..

[22] Daudt H.M.L., Van Mossel C., Scott S.J. (2013). Enhancing the Scoping Study Methodology: A Large, Inter-Professional Team’s Experience with Arksey and O’Malley’s Framework. BMC Med. Res. Methodol..

[23] Peters M.D.J., Marnie C., Tricco A.C., Pollock D., Munn Z., Alexander L., McInerney P., Godfrey C.M., Khalil H. (2020). Updated Methodological Guidance for the Conduct of Scoping Reviews. JBI Evid. Synth..

[24] Munn Z., Peters M.D.J., Stern C., Tufanaru C., McArthur A., Aromataris E. (2018). Systematic Review or Scoping Review? Guidance for Authors When Choosing between a Systematic or Scoping Review Approach. BMC Med. Res. Methodol..

[25] Levac D., Colquhoun H., O’Brien K.K. (2010). Scoping Studies: Advancing the Methodology. Implement. Sci..

[26] Tricco A.C., Lillie E., Zarin W., O’Brien K.K., Colquhoun H., Levac D., Moher D., Peters M.D.J., Horsley T., Weeks L. (2018). PRISMA Extension for Scoping Reviews (PRISMA-ScR): Checklist and Explanation. Ann. Intern. Med..

[27] Bramer W.M., de Jonge G.B., Rethlefsen M.L., Mast F., Kleijnen J. (2018). A Systematic Approach to Searching: An Efficient and Complete Method to Develop Literature Searches. J. Med. Libr. Assoc..

[28] Ouzzani M., Hammady H., Fedorowicz Z., Elmagarmid A. (2016). Rayyan—A Web and Mobile App for Systematic Reviews. Syst. Rev..

[29] Page M.J., McKenzie J.E., Bossuyt P.M., Boutron I., Hoffmann T.C., Mulrow C.D., Shamseer L., Tetzlaff J.M., Akl E.A., Brennan S.E. (2021). The PRISMA 2020 Statement: An Updated Guideline for Reporting Systematic Reviews. BMJ.

[30] Braun V., Clarke V. (2021). Thematic Analysis: A Practical Guide.

[31] Patton M.Q. Qualitative Research & Evaluation Methods|SAGE Publications Inc. https://us.sagepub.com/en-us/nam/qualitative-research-evaluation-methods/book232962.

[32] (2024). A Cura Della Redazione Indicators and Outcomes Associated with the Quality of Care: How to Use Them and Interpret Results. Assist. Inferm. Ric..

