# Supplementary material for: Characteristics, Antecedents, and Consequences of Non-Nursing Tasks: A Scoping Review Protocol
Source: Nurs Rep. 2025 Apr 30;15(5):153. doi: 10.3390/nursrep15050153 (PMC12114207; doi:10.3390/nursrep15050153)
Supplement: Supplementary file 1 [file nursrep-15-00153-s001.zip › nursrep-3580605-supplementary.pdf]

Table S1. Members of RICI Group

**University and Members**
University of Udine, Department of Medicine, Udine, Italy
Alvisa Palese, Stefania Chiappinotto, Alessandro Galazzi, Federico Fonda, Sara Dentice, Chiara Moreal, Gaia Dussi, Gaia Magro, Chiara Visintini, Erica Visintini
University of Torino, Department of Clinical and Biological Sciences, Italy
Valerio Dimonte, Paola Di Giulio, Sara Campagna, Beatrice Albanesi, Marco Clari, Alessio Conti, Silvia Gonella, Daniela Berardinelli, Elena Casabona, Federica Fenoglio, Federica Riva Rovedda, Elena Viottini, Jacopo Olagnero
University of Piemonte Orientale, Department of Translational Medicine, Novara, Italy
Alberto Dal Molin, Erika Bassi, Ines Basso, Erica Busca, Isabella Santomauro
University of Verona, Department of diagnostics and public health, Italy
Luisa Saiani, Federica Canzan, Elisa Ambrosi, Jessica Longhini, Giulia Marini, Michela Flippi, Arianna Caliaro
